# Rehab on Wheels: A Pilot Study of Tablet-Based Wheelchair Training for Older Adults

**DOI:** 10.2196/rehab.4274

**Published:** 2015-04-30

**Authors:** Edward Mark Giesbrecht, William C Miller, Boyang Tom Jin, Ian M Mitchell, Janice J Eng

**Affiliations:** ^1^Rehabilitation Research Program, Vancouver Coastal Health Research InstituteDepartment of Occupational TherapyUniversity of ManitobaWinnipeg, MBCanada; ^2^Rehabilitation Research Program, Vancouver Coastal Health Research InstituteDepartment of Occupational Therapy and Occupational ScienceUniversity of British ColumbiaVancouver, BCCanada; ^3^Department of Computer ScienceUniversity of British ColumbiaVancouver, BCCanada; ^4^Rehabilitation Research Program, Vancouver Coastal Health Research InstituteDepartment of Physical TherapyUniversity of British ColumbiaVancouver, BCCanada

**Keywords:** wheelchairs, telemedicine, self-efficacy, aged, pilot projects

## Abstract

**Background:**

Alternative and innovative strategies such as mHealth and eLearning are becoming a necessity for delivery of rehabilitation services. For example, older adults who require a wheelchair receive little, if any, training for proficiency with mobility skills. This substantive service gap is due in part to restricted availability of clinicians and challenges for consumers to attend appointments. A research team of occupational therapists and computer scientists engaged clinicians, consumers, and care providers using a participatory action design approach. A tablet-based application, Enhancing Participation In the Community by improving Wheelchair Skills (EPIC Wheels), was developed to enable in-chair home training, online expert trainer monitoring, and trainee-trainer communication via secure voice messaging.

**Objective:**

Prior to undertaking a randomized controlled trial (RCT), a pilot study was conducted to determine the acceptability and feasibility of administering an mHealth wheelchair skills training program safely and effectively with two participants of different skill levels. The findings were used to determine whether further enhancements to the program were indicated.

**Methods:**

The program included two in-person sessions with an expert trainer and four weeks of independent home training. The EPIC Wheels application included video instruction and demonstration, self-paced training activities, and interactive training games. Participants were provided with a 10-inch Android tablet, mounting apparatus, and mobile Wi-Fi device. Frequency and duration of tablet interactions were monitored and uploaded daily to an online trainer interface. Participants completed a structured evaluation survey and provided feedback post-study. The trainer provided feedback on the training protocol and trainer interface.

**Results:**

Both participants perceived the program to be comprehensive, useful, and easily navigated. The trainer indicated usage data was comprehensive and informative for monitoring participant progress and adherence. The application performed equally well with multiple devices. Some initial issues with log-in requests were resolved via tablet-specific settings. Inconsistent Internet connectivity, resulting in delayed data upload and voice messaging, was specific to individual Wi-Fi devices and resolved by standardizing configuration. Based on the pilot results, the software was updated to make content download more robust. Additional features were also incorporated such as check marks for completed content, a more consumer-friendly aesthetic, and achievement awards. The trainer web interface was updated to improve usability and provides both a numerical and visual summary of participant data.

**Conclusions:**

The EPIC Wheels pilot study provided useful feedback on the feasibility of a tablet-based home program for wheelchair skills training among older adults, justifying advancement to evaluation in an RCT. The program may be expanded for use with other rehabilitation interventions and populations, particularly for those living in rural or remote locations. Future development will consider integration of built-in tablet sensors to provide performance feedback and enable interactive training activities.

**Trial Registration:**

ClinicalTrials.gov NCT01644292; https://clinicaltrials.gov/ct2/show/NCT01644292 (Archived by WebCite at http://www.webcitation.org/6XyvYyTUf).

## Introduction

### Overview

Alternative and innovative electronic and mobile technology strategies are becoming increasingly important as platforms for delivery of health-related services [1]. Emergent research literature has demonstrated effective interventions for health literacy [2,3], self-management [4], and adherence and health behavior change programs [5]. However, mHealth has thus far been limited in its application to motor-skill training and rehabilitation services. Occupational and physical therapists often provide rehabilitation in a hospital setting. However, decreasing resources for continued outpatient rehabilitation has resulted in challenging and costly access, particularly for clients living in rural and underserviced communities [6,7]. The literature is beginning to document the benefits of using telehealth and mHealth as augmentative or alternative strategies to traditional in-person, individualized rehabilitation models [8]. In the previous decade, investigators explored in-home video telerehabilitation; however, this involved cumbersome camera equipment and coordination of real-time availability for client and clinician [9,10]. Near-ubiquitous Internet access and the emergence of lower-cost, portable, and powerful mobile devices such as smartphones and tablets have provided new opportunities for delivery of home-based rehabilitation.

One example of potential service delivery is the provision of wheelchair skills training, particularly among older adults. In Canada, there are an estimated 220,000 wheelchair users [11], over half of those being over the age of 65 [12]. Unfortunately, the growing numbers of older adults who require a wheelchair receive little if any training for proficiency with mobility skills [13,14]. This substantive service gap is due to restricted availability and time for clinicians to provide one-to-one therapy, limited content expertise, and challenges for consumers to attend appointments, particularly in rural or remote locations [15].

A tablet-based application, Enhancing Participation In the Community by improving Wheelchair Skills (EPIC Wheels), was developed to address this issue. The mobile device enables in-chair home training, asynchronous online expert trainer monitoring, and trainee-trainer communication via secure voice messaging. The EPIC Wheels content was developed using a social cognitive theory framework to optimize wheelchair-specific self-efficacy [16,17]. Self-efficacy has a demonstrated link to skill development and participation among wheelchair users [18]. Furthermore, incorporating self-efficacy strategies produces stronger adherence in home programs [19]. Four principal constructs promoting self-efficacy are integrated into EPIC Wheels content. *Mastery experience*, or the perception of performance achievement, is promoted by grading training activities from simple to complex to ensure early success experiences. Observing success in a comparable peer, or *vicarious experience*, is achieved by using age-appropriate models from both sexes for the demonstration videos. Personalized sessions and voice messaging contact with the trainer engenders *verbal persuasion* or reinforcement from a significant other. Finally, incorporating frequent but short training activities and self-monitoring exertion addresses participants’ reinterpretation of their *physiological state*. Principles from adult learning theory, or andragogy, [20,21] were used to structure the delivery of content with the EPIC Wheels program, as these have proven effective with mHealth behavioral interventions [6]. Andragogy theory proposes that adult learners are internally motivated and prefer to direct their learning, they bring life experience and knowledge to the learning process, they are goal-oriented, they desire learning that is relevant to their social role, they prefer practical learning strategies, and they like to be respected in the learning process. As a self-directed mHealth application, EPIC Wheels allows participants to negotiate their own home training schedule and navigate the program to work on the skills and functions that are most personally relevant and important.

The EPIC Wheels program was conceived as a three-phase project. Phase one involved design, evaluation, and revision of the training program content and method of delivery. Before undertaking a clinical trial, it is prudent to conduct a preliminary evaluation of the feasibility of study methods and procedures in a pilot study [22]. This paper reports on phase two, which is a pilot study focusing on administration and acceptability of the intervention processes to ensure components are well integrated and viable [23,24]. Once confirmed, phase three would be a randomized controlled trial (RCT) evaluating the impact of EPIC Wheels on wheelchair mobility skill among older adult novice manual wheelchair (MWC) users.

### Program Development and Content

Using a participatory action design approach [25,26], clinicians, consumers, and care providers engaged with occupational therapists and computer scientists on the study team to develop the EPIC Wheels program in phase one. Through an iterative process of design, evaluation, feedback, and revision, the program prototype progressed through three preliminary versions. A total of eight focus groups were conducted involving 34 participants from six stakeholder groups in two large urban centres. Focus group participants interacted with and critiqued each successful prototype, and the study team made evolving revisions until a beta version was ready for pilot testing in phase two. A detailed description of this development process has been reported in a previous publication [27]. As an extension to the participatory design process, participant feedback from the phase two pilot study further contributed to refinement of the EPIC Wheels program.

### Purpose and Objectives

As is often the case with rehabilitation interventions, there is considerable complexity evaluating EPIC Wheels due to the multiple components of administration, various behavioral requirements, and the tailored aspect of the program. The degree of clinical impact may be a consequence of program effectiveness or potentially an issue of implementation; therefore, process evaluation is critical. Best practice suggests that fidelity in the implementation protocol should be established and reported on using a pilot study as part of a systematic framework for evaluating complex interventions in clinical trials [28]. The intent of this pilot study was to run a preliminary version of the EPIC Wheels procedures to ensure integrity and integration of the study components, fidelity of the intervention protocol and methodological integrity [29], viability of participant adherence or engagement [30], and participant acceptance [31]. Rather than a *feasibility study*, which operates as a mini-RCT focusing on recruitment and primary outcome estimates, a *pilot study* addresses study-related issues of procedural administration, data collection, and intervention-specific issues [24]. Given the small scale, absence of a control group, and potential for changes based on the results, there was no intent to conduct hypothesis testing or include the data in the full clinical trial [23,24]. Thabane et al [23] propose the use of a framework for evaluation of process, resource, management, and scientific outcomes in a pilot study. Using this structure, we developed a comprehensive set of metrics by which to evaluate each component, including parameters for confirming feasibility. Consequently, the specific study objectives were to determine whether a wheelchair skills training program could be administered effectively and safely in an mHealth format, whether participants would adhere to the prescribed mHealth training protocol and find the training program acceptable and beneficial, and if additional changes or enhancements to the mHealth program were indicated.

## Methods

### Participants

Given the purpose of methodological evaluation, a sample size calculation was not indicated. Pilot studies typically involve a small sample, with 2-4 participants generally being sufficient to verify procedural feasibility [31]. We selected a purposive sample of two participants of different skill levels - one experienced and one novice MWC user. The experienced user (participant 1) would provide perspective on the applicability and relevance of the program and bring a larger spectrum of skills, enabling the trainer to anticipate how to adjust the training process accordingly. The novice user (participant 2) would be reflective of the target population. Participant 1 was a 60-year-old single male with a T9 spinal cord injury who had been a MWC user for 485 months and a competitive wheelchair athlete earlier in life. He was recruited through previous contact in phase 1 of the EPIC Wheels project, where he had expressed interest but was unable to participate in the program development. Participant 2 was a 73-year-old married male with left above-knee amputation who had been a MWC user for 3 months and was recruited through public advertisement. Both participants had home computers and a basic level of computer literacy but neither had a tablet device. Approval for the study was obtained from the University of Manitoba Health Research Ethics Board (#H2012:069) and registered with clinicaltrials.gov (NCT01644292). Participants completed a consent form that clearly articulated this was a pilot study to evaluate study procedures and participant acceptability.

### Study Overview

**Figure 1:**
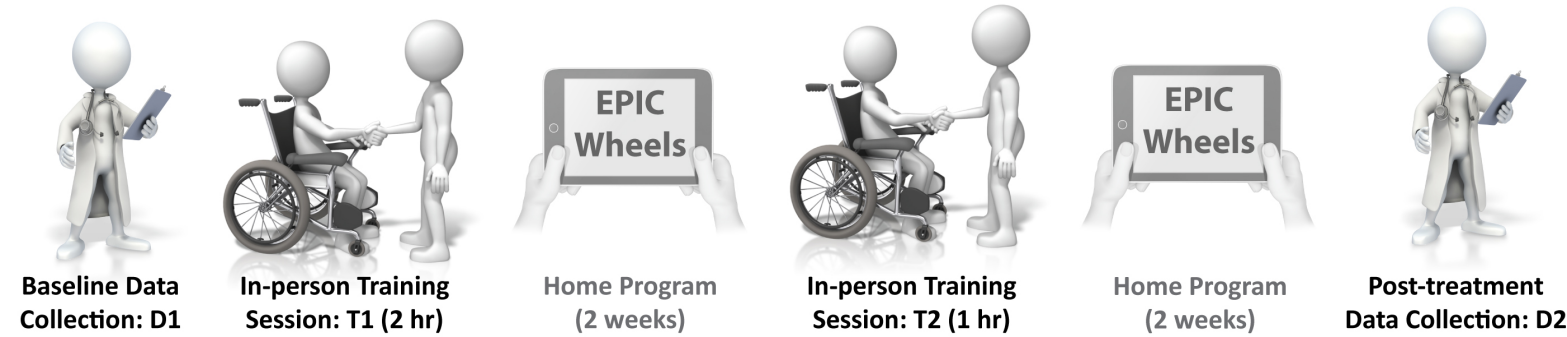
Study components and timeline.

Based on clinical consensus during the EPIC Wheels development phase, a four-week timeline was constructed to administer the program (see Figure 1). Acceptable time intervals for each milestone were identified in advance. Participants attended a baseline data collection appointment (D1) and then scheduled the first in-person training appointment (T1) within 7 days. After 14 days (optimal; must be between 12 and 16 days after T1) of home training with the tablet, participants attended a second in-person training session (T2). After another 14 days of home training, the program was complete and post-treatment data were collected (D2) within 42 days of D1. All data collection and in-person training occurred in a centrally located wheelchair-accessible clinic.

### Intervention Description

**Figure 2:**
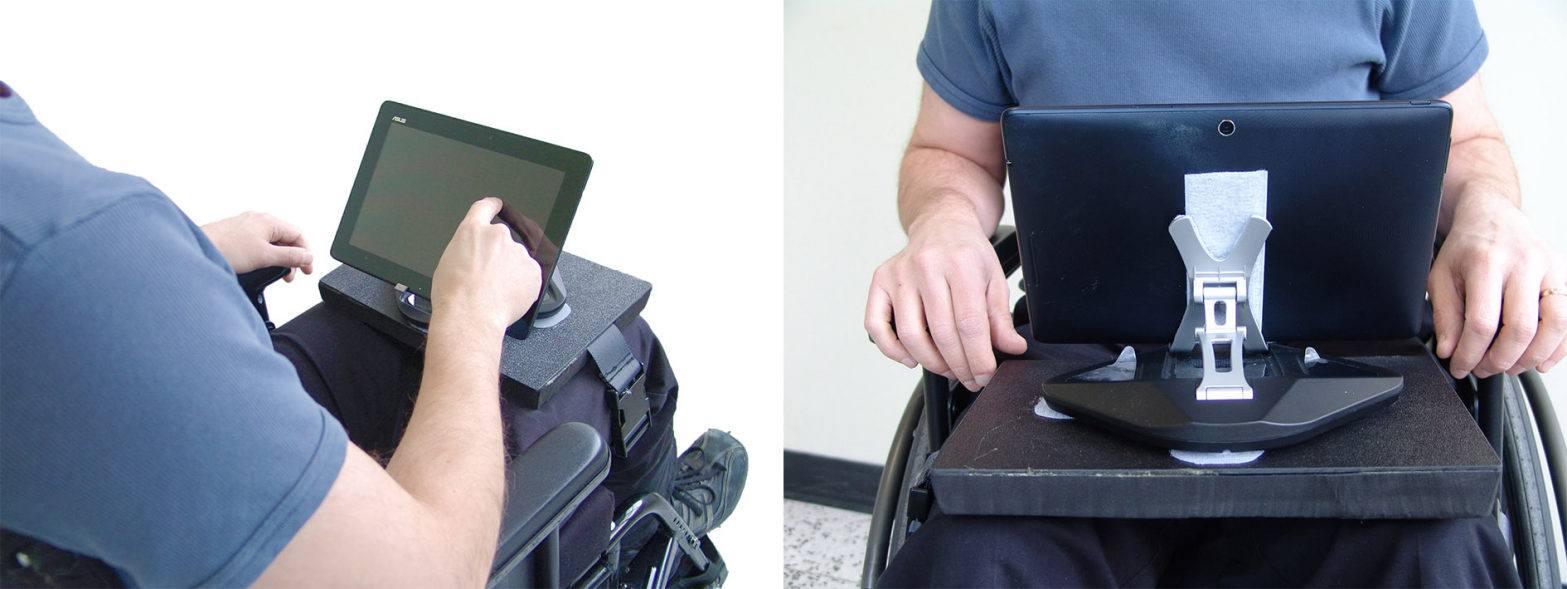
Tablet and mounting platform for in-chair training.

The EPIC Wheels program incorporates two brief in-person education sessions with an expert trainer and four weeks of monitored home training conducted via a computer tablet. The first education session involves one hour of individualized assessment of specific mobility-related wheelchair skills and one hour of orientation to the tablet and software program. The trainee is provided with a password-protected 10” Android tablet configured for single-function use (ie, only the EPIC Wheels program is accessible) along with a pre-synchronized mobile Wi-Fi device to provide Internet access. We intentionally used two different tablets (Motorola XOOM and ASUS TF300) and mobile Wi-Fi devices (Huawei E587 and Sierra AirCard 763S) to ensure a spectrum of device compatibility and functionality. A tablet stand mounted on a cushioned platform rests on the trainee’s lap, secured in place with a strap around the thighs for in-chair use (see Figure 2).

The tablet home program incorporates a variety of training components provided in video format. Participants view videos from one to five minutes in length that provide education and demonstration of specific wheelchair mobility skills. Additional videos require participants to practice demonstrated skills for a prescribed period of time using an on-screen timer with a start/stop function. Other videos incorporate interactive games and activities that require participants to perform maneuvers in response to or synchronous with the displayed video content. The training videos are structured to encourage repetition and variation of skill performance consistent with motor learning principles. Skills are broken down into subcomponents and progress from simple to complex. The initial section contains five chapters beginning with detailed information and instruction related to safety, injury prevention, and caregiver spotting; subsequent sections are locked out until the safety section is completed. The remaining four sections cover wheelchair components and body positioning; propulsion strategies; basic skills, such as turning around and negotiating obstacles; and advanced skills, such as ascending and descending thresholds and inclines, crossing gaps and soft surfaces, negotiating doorways, and managing curbs and stairs.

Trainees are instructed to practice at home 4 to 5 days per week in 15-30 minute sessions for a total of at least 75 minutes each week. All tablet activity is internally recorded and uploaded to a secure server which the trainer can access online. Two prompting questions are posed when the trainee engages the program (questions only appear once per 24 hours), requiring responses. The first question asks “*Did you have any tips or falls?*” If the response is yes, trainees receive an additional prompt to contact their trainer. The second question asks “*Since your last session, did you do any training on your own?*” If trainees select yes, they receive an additional prompt to select the number of minutes spent practicing without the tablet in 5-minute increments. Trainer and trainee can exchange voice messages from their respective computers and tablets at their convenience. Based on the monitored data, the trainer may initiate contact if concerns arise (ie, if there is no training activity for 2-3 days) or adapt the content of the second education session (ie, if the trainee is advancing quickly through the progression of skills). After two weeks of home training, the trainee attends a second in-person education session of 1 hour in length. The trainer reviews home program activities and provides additional, more advanced skills training, and the trainee continues with the EPIC Wheels home program for another two weeks.

As there are inherent safety risks with wheelchair use, primarily related to tips and falls, several safety strategies were employed. Participants were encouraged to bring a care provider to the in-person training sessions and have them supervise higher-risk training activities at home. Safe spotting and supervision instruction were provided at the first training session along with a spotter’s strap (to prevent rearward tips) for home use.

### Data Collection and Analysis

Dates for completion of each study component were documented and intervals calculated. The study tester administered D1 and D2 in accordance with a detailed protocol binder and corresponding checklist. The first author confirmed procedural and scoring accuracy via video recordings; any discrepancies or errors were reviewed with the tester and additional training provided if necessary. If procedural issues arose, these were documented and protocols modified. The principal clinical outcomes of the intervention were wheelchair skill capacity and safety as measured by the Wheelchair Skills Test (WST 4.1) [32], as well as wheelchair-specific self-efficacy as measured by the Wheelchair Use Confidence Scale (WheelCon-M 3.0) [33]. The WST is a standardized, performance-based measure of 32 skills, each evaluated on a capacity subscale and a safety subscale scored from 0 to 32. The WheelCon-M is a 65-item questionnaire in which respondents rate their confidence using a wheelchair in varying activities and environments on a scale from 0 (not confident) to 100 (completely confident), producing a mean confidence score between 0 and 100.

The study trainer administered T1 and T2 in accordance with a detailed protocol binder and corresponding checklist, with the first author again confirming accuracy via video recordings and addressing issues with the trainer or revising the protocol. The study trainer completed a post-treatment evaluation form and interview with the first author.

The EPIC Wheels software documented all tablet interactions with a time stamp and uploaded this data to the trainer website on a secure server. Training activity data (in minutes) were tabulated for each day and imported into an Excel spreadsheet. From this data, we were able to calculate the total number of days and minutes of training, mean number of days per week training, minutes per week training, and minutes per training day. Responses to the daily safety question prompt *“Did you have any tips or falls?”* were also recorded. When technical issues arose with the tablet or mobile Wi-Fi device, trainees contacted their trainer via the tablet voice-messaging feature. If the trainer was unable to resolve the issue, the first author traveled to the trainee’s home to troubleshoot the problem and document how it was resolved. Based upon the data analysis and feedback from trainer and trainees, the development team explored any further changes or revisions that could improve functionality or feasibility of the program.

After finishing all data collection at D2, trainees completed a 9-item post-treatment questionnaire evaluating elements critical to rehabilitation intervention development [34,35] on a Likert scale from 1 (strongly disagree) to 4 (strongly agree). Following this, the first author conducted an exit interview to obtain additional qualitative feedback about participant experiences. The interviews were conducted in a semistructured format and were 15 to 20 minutes in length. The sessions began with open-ended queries related to overall impressions of the program and then, following the participant’s lead, more focused questions were asked to elicit details about factors that enhanced or detracted from the training experience. Follow-up questions targeted specific impacts of the program on wheelchair use, impressions of the user interface, and perceptions of the program’s benefit. The first author took detailed notes during the interview and further refined details immediately afterwards. In addition, participant 1 shared written feedback related to program content, which he had brought to the D2 session.

The study trainer completed a post-treatment questionnaire after finishing with each participant which included five dichotomous questions (yes/no) related to clarity, timeliness, and issues with and major/minor deviations from the intervention protocol, with the option for narrative explanation. The first author also conducted an informal exit interview with the trainer after participant 2 had finished the study. The interview was approximately 15 minutes long and employed an unstructured format. The trainer was invited to share her experience with the training intervention and explicate both benefits and shortcomings. Follow-up questions were spontaneous and intended to elicit additional detail or clarification. Experience with and impressions of the monitoring website were one area of specific exploration. General notes were taken during the interview and additional detail constructed immediately afterwards by the first author.

## Results

All study components were completed within the prescribed time allocations. Administration of the data collection and in-person training sessions were consistent with protocol guidelines, with minor revisions (see Multimedia Appendix 1). No adverse events were encountered during any data collection or training sessions. The principal clinical outcomes of wheelchair skill capacity and safety as well as wheelchair-specific self-efficacy are presented in Table 1. Participant 1 (the experienced MWC user) demonstrated no change in wheelchair skill and safety, but his self-efficacy score increased by 5.9 (5.9%). Participant 2 (the novice MWC user) had improved scores in skill capacity (12.5%), safety (3.2%), and self-efficacy (7.2%).

**Table 1:** Wheelchair skill capacity, safety, and self-efficacy scores.

Measure	Participant 1		Participant 2	
	Baseline	Post-intervention	Baseline	Post-intervention
WST: capacity (%)	24 (75.0)	24 (75.0)	18 (56.3)	22 (68.8)
WST: safety (%)	32 (100)	32 (100)	29 (90.6)	30 (93.8)
WheelCon-M	79.3	85.2	63.9	71.1

With respect to adherence with tablet home program expectations, the frequency of training (days spent training each week) was 4, 3, 4, and 4 (total 15 days) for participant 1 and 6, 5, 3, and 6 (total 20 days) for participant 2. The intensity of training sessions (mean minutes per training day) was 36.9 minutes for participant 1 and 30.4 minutes for participant 2. In terms of training dosage, participant 1 spent a total of 553 minutes in home training (138.3 minutes/week) while participant 2 spent a 608 minutes training with the tablet (152.0 minutes/week). Neither participant reported any adverse events or injuries during home training.

A summary of participant responses to the post-treatment questionnaire is detailed in Table 2. During the post-treatment interview, participant 1 indicated the program was excellent and would have been beneficial to him during his initial transition to wheelchair use. He stated the training activities were fun and engaging, some of which he had modified on his own to increase the complexity and challenge given his existing level of skill proficiency. One observation he made was the uncertainty around how far he was through a given training video. Videos were limited to play, pause, and stop functions, and the participant didn’t know how much running time had passed or was remaining. Participant 2 reported a number of areas of specific skill improvement including propelling over high resistance surfaces and maneuvering around corners. He highlighted the comfort and ease he now had with “popping his casters” to get over small obstacles in his home and community and reflected on how this had seemed an impossibility to him during the baseline assessment.

**Table 2:** Post-treatment questionnaire responses by participant 1 and 2.

Item	Strongly agree	Agree	Disagree	Strongly disagree
Training is valuable or important	P1^a^, P2^b^			
Method of training was reasonable and appropriate	P1	P2		
Skills taught were reasonable and appropriate	P1	P2		
Trainer was reasonable and appropriate	P1, P2			
Expectations were manageable and practical		P1, P2		
Components of program provided as described	P1	P2		
I was able to perform or improve skills taught	P1	P2		
I did not experience injury or undue physical/mental stress	P1	P2		
Program was successful in improving my skills	P2	P1^c^		
Total	10	8	0	0

^a^P1: Participant 1.

^b^P2: Participant 2.

^c^This participant self-modified some of the activities to increase the challenge/difficulty.

The trainer indicated no major/minor deviations or issues with administering the intervention and confirmed satisfactory timeliness and clarity of process with both participants. At T1, set-up of the Wi-Fi device occurred after the tablet program orientation; consequently, the trainer was unable to demonstrate the daily prompting questions, which proved to be problematic for the participants. During the exit interview, the trainer highlighted the value of being able to monitor participant training activities online to identify potential problems (ie, no training activities for several days) and adapt the intervention content and goals based on participant progress. However, the trainer identified that data was collated into daily totals and did not explicate multiple sessions within a given day. In addition, the details of training activity (ie, specificity and frequency of which components participants engaged in) were not available. These shortcomings were identified as a limitation to capturing a full picture of participant training activity. Participant 2 reported several occasions when the voice message function failed to send and receive messages, compelling him to contact the trainer via telephone. The trainer also identified extended time periods between participant practice data uploading to the website. This also proved to be frustrating for the participant because his training time was not included in the progress window. The first author traveled to the participant’s home on two occasions before diagnosing an issue with the Wi-Fi timing out, resulting in the tablet losing Internet connectivity. Revision of the tablet and Wi-Fi configuration settings resolved these issues.

## Discussion

### Evaluation of Program Administration, Adherence, and Acceptability

The results of the pilot study demonstrated that, with several minor revisions, the EPIC Wheels RCT could feasibly be administered as planned. With respect to the administration of the data collection and treatment intervention procedures, these were conducted efficiently within the proposed timeframe of six weeks and consistently within the outlined protocols. While no breaks from protocol were encountered during in-person training sessions, a revision to the T1 session was instituted as a result of the pilot experience. Wi-Fi connectivity is now initiated prior to the tablet orientation to ensure the daily prompting questions appear, allowing the trainer to demonstrate this feature. The trainer also confirmed feasibility of the intervention protocol, including the sufficiency of a 1-hour orientation to the tablet and EPIC Wheels software program with novice users. The trainer website provided useful and relevant data for basic monitoring of trainee progress; however, the trainer identified that additional detail about the specificity of training activities and multiple daily sessions would be desirable. The voice-messaging issue proved to be frustrating for the trainer and trainee because it required coordinating a contact time via telephone. Delays in data upload to the website were concerning as the trainer could not ascertain whether the participant was not actually engaging in any training or whether this data were simply not being reported. Participant 2 identified the progress window as highly motivating and was upset when completed practice was not recognized. Even small issues such as these with an mHealth user interface could potentially compromise usage and adherence, and this experience was valuable in highlighting the benefits of pilot testing to resolve any issues with seamless delivery.

Viability of the EPIC Wheels program with multiple Android tablet and mobile Wi-Fi device combinations was confirmed. Intermittent connectivity issues with the mobile Wi-Fi devices required troubleshooting during the home training component of the study until a satisfactory configuration was obtained. As a result, tablet/Wi-Fi device specifications were documented to optimize setup for future participants. In addition, printed user guides were created for each tablet/Wi-Fi device combination. Ideally, using a participant’s home Wi-Fi would eliminate most potential connectivity problems as well as the cost of renting a mobile Wi-Fi device (approximately $10/month). However, this requires configuration of tablet settings in-home, which can present several barriers. First, the tablet is configured as a single application device preventing participants from accessing other applications or tablet settings. This restriction can be overridden but would require that a study administrator either visit the participant’s home and make these adjustments (potentially requiring participants to reveal a security password) or convey these procedures to the participant. The latter option would necessitate the participant or surrogate possesses the capacity to operationalize the changes or study personnel to provide continuing technical support from a distance and would increase the potential for additional untoward modifications or alternate use of the tablet. Second, home Wi-Fi availability is not ubiquitous, particularly among the target population of older adults. A recent survey estimates that in the United States, only 47% of seniors have high-speed Internet connectivity in their home [36]. An alternative solution would thus be required for individuals without Internet access and for those with connectivity but no Wi-Fi service.

An important objective of this study was to ensure the expectations of the home training program were reasonable and safe and that participant adherence was feasible. Without confirming these elements, valid evaluation of the intervention as intended in the subsequent RCT would be in jeopardy. Both participants met or exceeded the targeted parameters of adherence; participant 1 was slightly under the desired frequency of days practicing but exceeded the minimum session intensity and dosage metrics. Both participants spent nearly twice the minimum recommended time engaged with the mHealth platform, training a total of approximately 10 hours over four weeks. Frequency of practice is a critical component in developing motor skills [37] and using a mobile tablet application rather than a Web-based program accessible only via computer offers greater flexibility to encourage multiple training sessions in varied contexts [2]. Participant 1 had fewer practice sessions and spent slightly less time overall with the home program. However, given his level of proficiency with wheelchair use he may have been less motivated to engage in watching and practicing skills he had already mastered. Neither participant reported any adverse events, including tips or falls; each agreed they did not experience undue mental or physical stress and the program methods and expectations were reasonable.

In addition to confirming the EPIC Wheels program was reasonable and safe, participant acceptability and perception of program relevance and benefit was paramount. While evaluation of clinical outcomes was not the primary purpose, the results from the pilot study were promising. Participant 1 was an expert MWC user and, as expected, did not improve in skill capacity or safety. However, he did show a small improvement in self-efficacy even after 40 years of experience. Participant 2, who was a novice user and representative of the target population, demonstrated improvements in skill capacity, safety, and self-efficacy. The improved wheelchair skill scores suggest that the EPIC Wheels intervention could be effective in achieving the desired outcome. Furthermore, the improvement seen in self-efficacy among both participants supports the theoretical basis of the training program using social cognitive theory constructs. Current evidence suggests that, in addition to wheelchair skill capacity, higher self-efficacy is positively associated with frequency of participation among older wheelchair users [38].

With respect to the trainee post-treatment questionnaire, our evaluation metric was to have both participants agree or strongly agree with each item, which was confirmed. Both participants confirmed the content was appropriate and beneficial. Both participants described the mHealth platform as engaging and entertaining, as well as providing an appropriate context and delivery strategy for learning new wheelchair skills. These positive evaluations regarding the EPIC Wheels intervention appear reasonable, given that both participants experienced improvement in self-efficacy and the novice MWC user also increased his capacity and safety with wheelchair use. Since most telerehabilitation and mHealth interventions target behavioral or cognitive skills and strategies, this pilot study was particularly useful in providing initial evidence to support mHealth application to motor skill improvement.

### Changes and Enhancements to the EPIC Wheels User Interface

**Figure 3:**
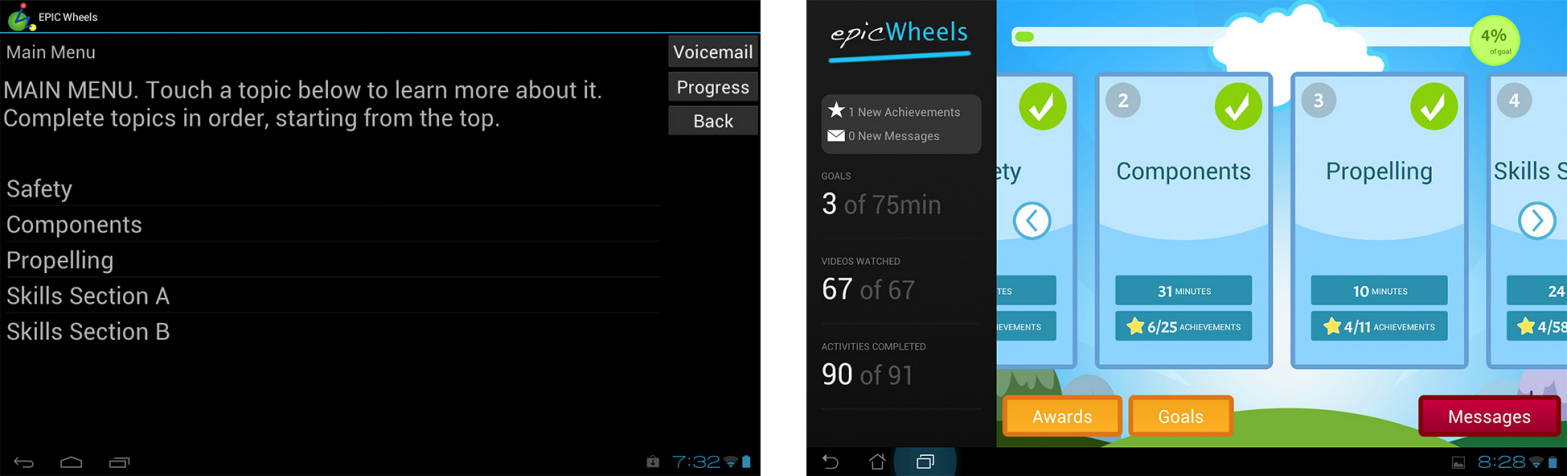
Trainee interface pre- and post-pilot versions.

**Figure 4:**
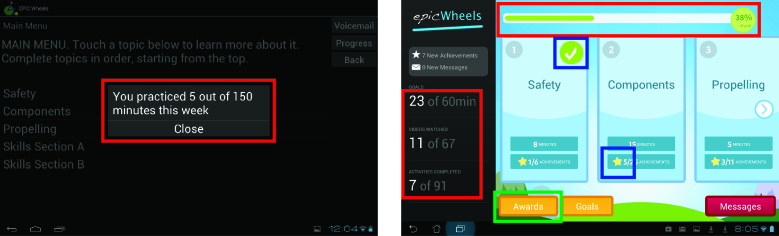
Participant progress display pre- and post-pilot versions.

**Figure 5:**
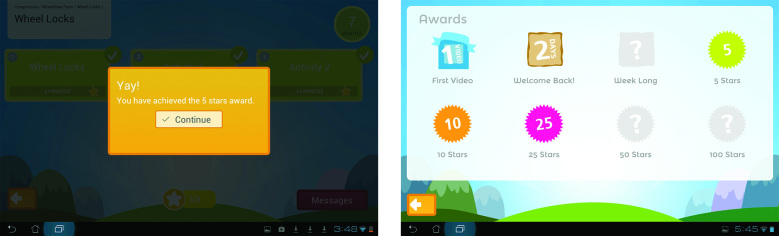
Award pop-up with Awards Earned windows in post-pilot version.

While neither trainee identified overwhelming concerns with the user interface, conveying participant practice data and progress was an issue for both trainee and trainer. Improved navigation of the program and individual training videos were also identified as desirable. While both participants rated all components of the post-treatment questionnaire as at least satisfactory, the study team felt that additional information and improved aesthetics could further enhance adherence and usability in the subsequent RCT, which would be reflected in future evaluations. Consequently, several modifications were made to the home program. The user interface was upgraded with a more colorful and dynamic appearance, consistent with other consumer applications (Figure 3). Participant progress information is in constant display, rather than opening in a new window, and includes not just the number of minutes practiced but the number of instructional videos viewed and activities completed as well as a progress bar for the current training week (Figure 4, red highlights). When completed, training components now display a visual check mark (to simplify navigation to the current training activity) and a gold star (Figure 4, blue highlights). The gold stars cumulatively earn progress awards, which are delivered to the participant and can be viewed in a dedicated awards window (Figure 4, green highlight, and Figure 5).

**Figure 6:**
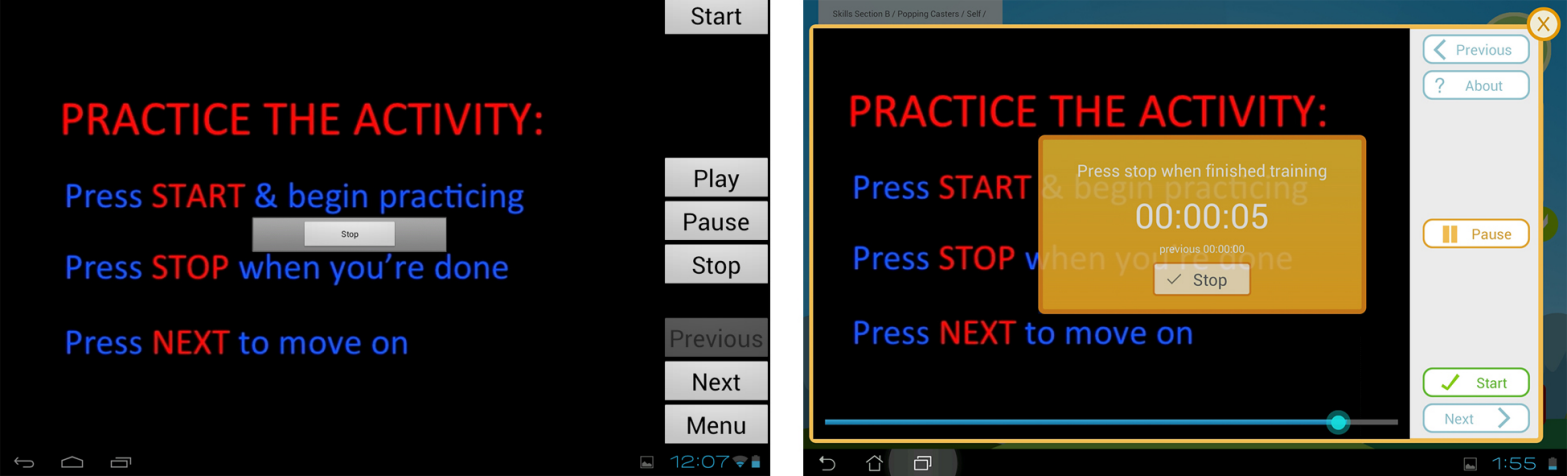
Training activity window with timer pre- and post-pilot versions.

The window for displaying training information and activities was modified to improve appearance and navigation (Figure 6). In particular, a scrubber bar was introduced to identify progress through the activity and allow trainees to easily navigate forward and backward. For timed training activities, the monochrome Start/Stop button was replaced with a larger, colorful button with more detailed directions and a clock with running time. The study team anticipates these modifications will provide better visibility and comprehension for older adult users and promote greater adherence to the suggested training time.

**Figure 7:**
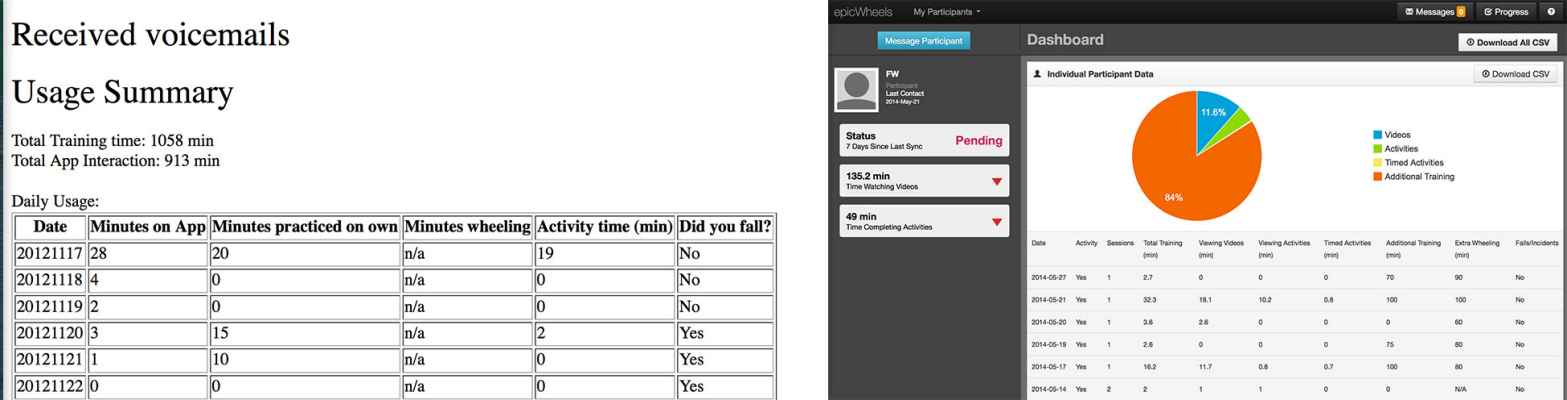
Trainer website pre- and post-pilot testing.

**Figure 8:**
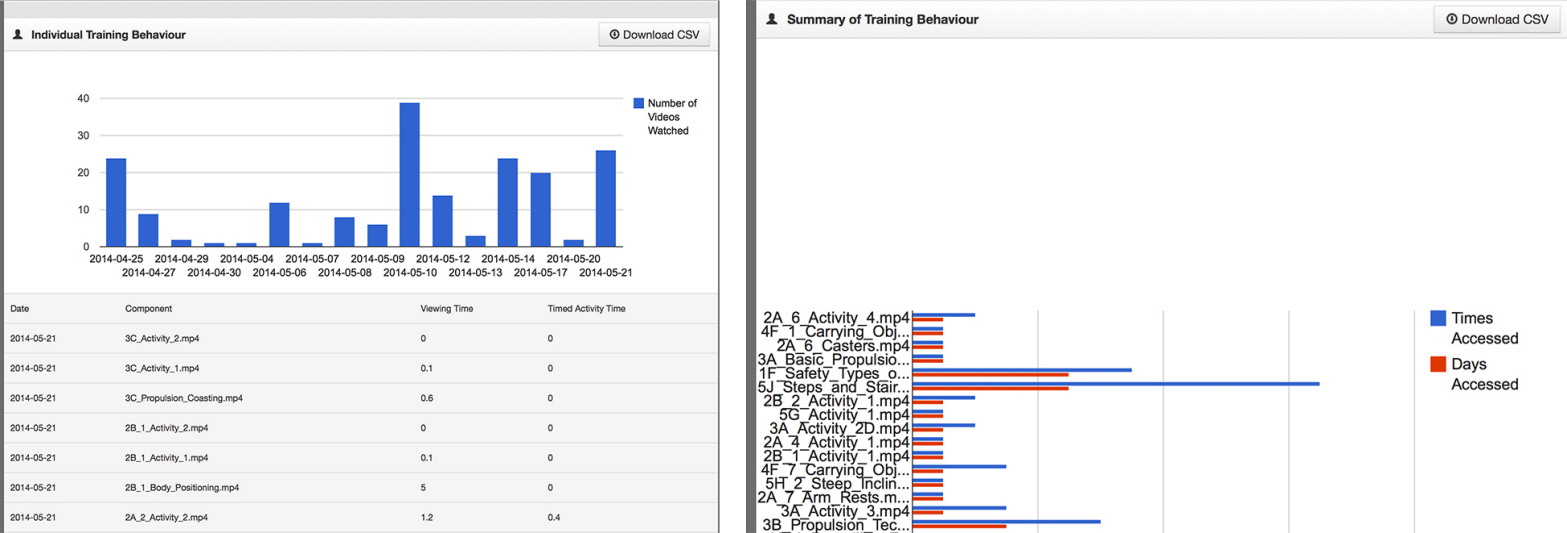
Trainer website features.

Based on suggestions from the trainer and discussion among the study team, the format and content of the trainer website was also modified to improve usability and appearance. The original site displayed a simple table with only the total minutes spent engaged in tablet activity on active training days as well as a running total (see Figure 7). The revised site now displays multiple training sessions on a given day in table format and a quick view graphic breakdown for the types of training done (eg, viewing educational videos, engaging in training activities, practice without the tablet). By scrolling down the page, the trainer can view additional graphic and tabular data explicating trainee usage for each home-training session (see Figure 8). The number of days accessed, time accessed, total views, length of time viewing, and associated time practicing is now available for each training component.

**Figure 9:**
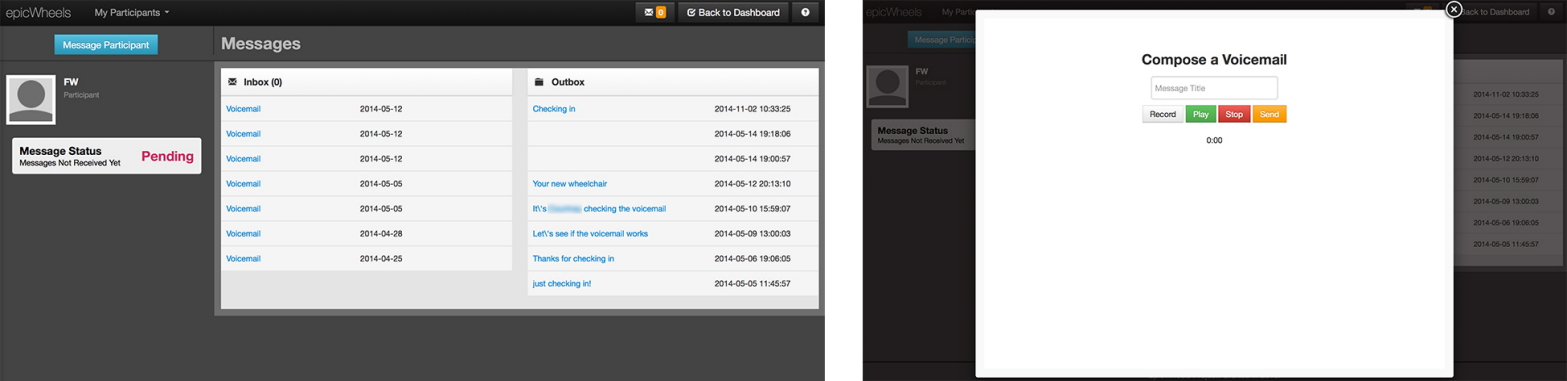
Trainer voice-messaging applet.

The voice-messaging software was restructured to use a more robust commercial application that does not require extensive configuration to the trainer’s computer and now provides efficient and reliable performance. The trainer website was also revised to incorporate a simple and intuitive voice-message user applet that also includes the option of a subject line (see Figure 9).

### Limitations

This pilot study provided sufficient confirmation of the fidelity of study procedures to proceed with a feasibility RCT. The small number of participants may have limited the scope of issues identified in the implementation and acceptability of the mHealth intervention. The investigators developed the evaluation structure and questionnaires with specificity to address usability and implementation issues of concern; however, the use of validated evaluation formats and measures would enhance the generalizability of results, and future studies should endeavor to employ them. The first author conducted the post-intervention interviews with participants, and they may have been reluctant to express concerns or criticism because of the relationship established during the study. A more extended interview with a structured guide or a series of interviews throughout the pilot study might have elicited additional information related to program attributes and factors contributing to success. Participant 2 subsequently provided a separate interview with a public access television station and expressed a comparably positive evaluation of the EPIC Wheels program [39].

### Conclusions

The EPIC Wheels pilot study provided confirmation of the feasibility of our study design to evaluate a tablet-based home program for wheelchair skills training among older adults. Participants reported positive impressions of the intervention and delivery method and the initial treatment effect results are promising. Feedback from participants and trainers resulted in several adaptations to the intervention, including expansion and upgrade of the user interface for both trainee and trainer. Effectiveness of the EPIC Wheels program will be evaluated in an RCT [40]. The program offers considerable potential for expansion and use with various populations and delivery of other rehabilitation training programs, particularly for those living in rural/remote locations having limited access to rehabilitation services, including those in developing nations. Future development will consider integration of built-in tablet sensors to provide performance feedback and enable interactive training activities.

## Multimedia Appendix 1

Data on administration of data collection and intervention.

## Abbreviations

EPIC Wheels: Enhancing Participation In the Community by improving Wheelchair Skills
MWC: manual wheelchair
RCT: randomized controlled trial
