# Supplementary material for: Rehab on Wheels: A Pilot Study of Tablet-Based Wheelchair Training for Older Adults
Source: JMIR Rehabil Assist Technol. 2015 Apr 30;2(1):e3. doi: 10.2196/rehab.4274 (PMC5454553; doi:10.2196/rehab.4274)
Supplement: Supplementary file 1 [file rehab_v2i1e3_app1.pdf]

Data for administration of the study components

Event Interval	Evaluation Metric	Participant 1	Participant 2	Outcome
D1 – T1	≤ 7 days	5	6	Confirmed
T1 – T2	14 ± 2 days	14	13	Confirmed
T1 – D2	28 - 35 days	28	29	Confirmed
D1 – D2	≤ 42 days	33	35	Confirmed

Data for administration of the data collection procedures

Component	Evaluation Strategy	Evaluation Metric	Outcome
Administration consistent with protocol binder	D_1 and D_2 video recorded; reviewed within 3 days	Any errors or issues noted and addressed or protocol revised	Confirmed
	Protocol checklist	All items completed and checked	Confirmed
Administration of outcome measures	Measures re-scored by PI via video recording	Errors or issues noted; addressed with tester or additional training	Confirmed
Administration burden	Tester documented time to administer; PI confirmed via video recording	D_1: 120 ± 10 min<br>P1 = 122 min; P2 = 114 min<br>D_2: 60 ± 10 min<br>P1 = 51 min; P2 = 69 min	Confirmed[a]

[a] Modification made to the D1 protocol to administer the primary outcome (WST) once, rather than twice, to reduce burden and administration time.

Data for administration of the in-person training procedures

Component	Evaluation Strategy	Evaluation Metric	Outcome
Administration consistent with protocol binder	T_1 and T_2 video recorded; reviewed within 3 days	Any errors or issues noted and addressed or protocol revised	Confirmed
	Protocol checklist	All items checked and completed	Confirmed
	Breaks from protocol	No breaks, else protocol revision	Confirmed
Administration burden	Trainer documented time to administer; PI confirmed via video recording	T_1: within 120 ± 10 min<br>P1 = 123 min; P2 = 101 min<br>T_2: within 60 ± 10 min<br>P1 = 60 min; P2 = 70 min	Confirmed
Safety	Adverse events reported	No adverse events	Confirmed
Trainer Acceptability	Post-T_2 Questionnaire:<br>- Major or minor deviations<br>- Protocol is clear<br>- Reasonable time available<br>- Issues with protocol	 NO (or revise protocol)<br>YES (or revise protocol)<br>YES (or revise protocol)<br>NO (or revise protocol)	Confirmed
